# Klimaanpassung im Mehr-Ebenen-System des Rechts

**DOI:** 10.1007/s10357-022-4055-z

**Published:** 2022-08-16

**Authors:** Johannes Saurer

**Affiliations:** Lehrstuhl für Öffentliches Recht, Umweltrecht, Infrastrukturrecht und Rechtsvergleichung, Universität Tübingen, Tübingen, Deutschland

## Abstract

Im Zentrum der Rechtsentwicklung im Klimaschutzrecht steht aktuell die Mitigation, also die Verringerung der Treibhausgasemissionen zur Stabilisierung der Erdatmosphäre. Es wird aber immer deutlicher, dass auch die Adaptation, d.h. die Anpassung an die Folgen der anthropogenen Erderwärmung eine dauerhafte Aufgabe des Rechts ist. Dieser Aufsatz untersucht im Überblick, in welchem Umfang sich auf den verschiedenen Ebenen des Rechts bereits Regelungen zur Anpassung herausgebildet haben und welche weiteren Regelungen in der rechtspolitischen Diskussion sind.

## 1. Klimaänderungen infolge der anthropogenen Erderwärmung und nachteilige Auswirkungen

Die anthropogene Erderwärmung infolge einer Überkonzentration von Treibhausgasen in der Atmosphäre führt schon heute weltweit zu signifikanten Klimaänderungen. Die durchschnittliche globale Erdoberflächentemperatur hat sich gegenüber vorindustrieller Zeit inzwischen um mehr als 1 Grad Celsius erhöht und nähert sich der 1,5 Grad Celsius-Marke.[1] Nach dem aktuellen Sechsten Sachstandsbericht 2021/2022 des Weltklimarats IPCC wirkt sich der „vom Menschen verursachte Klimawandel bereits auf viele Wetter- und Klimaextreme in allen Regionen der Welt aus".[2] Gegenüber dem vorhergehenden Sachstandsbericht 2014/2015 gebe es „stärkere Belege für beobachtete Veränderungen von Extremen wie Hitzewellen, Starkniederschlägen, Dürren und tropischen Wirbelstürmen sowie insbesondere für deren Zuordnung zum Einfluss des Menschen".[3] Der IPCC betont zudem die Langfristigkeit dieser Effekte: „Viele Veränderungen aufgrund vergangener und künftiger Treibhausgasemissionen sind über Jahrhunderte bis Jahrtausende unumkehrbar, insbesondere Veränderungen des Ozeans, von Eisschilden und des globalen Meeresspiegels."[4] Die EU-Kommission verzeichnet für die EU zunehmend Klima- und Wetterextreme, die „von nie da gewesenen Waldbränden über Hitzewellen nördlich des Polarkreises bis hin zu verheerenden Dürren im Mittelmeerraum, von vernichtenden Wirbelstürmen in Gebieten in äußerster Randlage der EU bis hin zu massiven Waldschäden infolge eines nie da gewesenen Borkenkäferbefalls in Mittel- und Osteuropa [reichen]".[5]

Das Umweltbundesamt hat unter Einbeziehung zahlreicher weiterer Stellen der Bundesverwaltung die umfassende Klimawirkungs- und Risikoanalyse 2021 für Deutschland vorgelegt.[6] Die Studie arbeitet mit einem Spektrum möglicher Erhöhungen der Temperatur in Deutschland gegenüber vorindustrieller Zeit zwischen 2,4 und 3 Grad bis zur Mitte des 21. Jahrhunderts. Zusammenfassend heißt es, es bestünden „besonders hohe Klimarisiken" für Böden, Wälder und Gewässer und Wirtschaftssysteme, die auf diese Ressourcen angewiesen sind und besonders vulnerabel für Folgen von Trockenheit sind (Fischerei, Land- und Forstwirtschaft).[7] Weitere Risiken seien die langsame Verschiebung von Anbauregionen, das Auftreten von neuen Schädlingen und eine Veränderung des Artenspektrums von Tieren und Pflanzen mit der möglichen Folge eines Artensterbens und Biodiversitätsverlusten.[8] Aspekte des Klimawandels wie verschlechterte Luftqualität und Hitzewellen hätten negative Folgen für die menschliche Gesundheit. Klimawandelfolgen wie Starkregen und damit verbundene Überschwemmungen bedrohten Gebäude, Industrieanlagen und Verkehrswege.[9] Der in der Folge der Erderwärmung zu erwartende weitere Anstieg des Meeresspiegels an den deutschen Küsten gilt als langfristig sehr großes und mehrdimensionales Risiko.[10]

## 2. Kontextgebundenheit und Vielgestaltigkeit von Anpassungsbedarf und -instrumenten – Normative Festlegung von Anpassungsszenarien

Die erheblichen – und in ihren Erscheinungsformen sehr unterschiedlichen – nachteiligen Auswirkungen der Klimaänderungen können im jeweiligen räumlichen, sozialen und infrastrukturellen Kontext sehr unterschiedlichen

Prof. Dr. Johannes Saurer, LL.M. (Yale),
Inhaber des Lehrstuhls für Öffentliches Recht, Umweltrecht, Infrastrukturrecht und Rechtsvergleichung, Universität Tübingen, Tübingen, Deutschland

1) Vgl. World Meteorological Organization (WMO), Pressemitteilung v. 9.5.2022, Stand 13.5.2022, abrufbar unter https://public.wmo.int/en/media/press-release/wmo-update-5050-chance-of-global-temperature-temporarily-reaching-15%C2%B0c-threshold.
2) Sechster IPCC-Sachstandsbericht (AR6), Beitrag von Arbeitsgruppe I: Naturwissenschaftliche Grundlagen, Version v. 29.10.2021, Hauptaussagen, Ziff. A.3, Stand 21.3.2022, abrufbar unter https://www.de-ipcc.de/media/content/Hauptaussagen_AR6-WGI.pdf.
3) Sechster IPCC-Sachstandsbericht (AR6), Beitrag von Arbeitsgruppe I: Naturwissenschaftliche Grundlagen, Version v. 29.10.2021, Hauptaussagen, Ziff. A.3, Stand 21.3.2022, abrufbar unter https://www.de-ipcc.de/media/content/Hauptaussagen_AR6-WGI.pdf.
4) Sechster IPCC-Sachstandsbericht (AR6), Beitrag von Arbeitsgruppe I: Naturwissenschaftliche Grundlagen, Version v. 29.10.2021, Hauptaussagen, Ziff. A.3, Stand 21.3.2022, abrufbar unter https://www.de-ipcc.de/media/content/Hauptaussagen_AR6-WGI.pdf.
5) Komm., Mitt. v. 24.2.2021, Ein klimaresilientes Europa aufbauen – die neue EU-Strategie für die Anpassung an den Klimawandel, COM(2021) 82 final, S. 1.
6) Klimawirkungs- und Risikoanalyse (KWRA) 2021 für Deutschland, Kurzzusammenfassung, Stand 21.3.2022, abrufbar unter https://www.umweltbundesamt.de/sites/default/files/medien/2546/dokumente/kurzzusammenfassung_kwra_2021_.pdf; vgl. auch ausführlich die Beiträge in *Brasseur/Jacob/Schuck-Zöller* (Hrsg.), Klimawandel in Deutschland, 2017, 77 ff.
7) Klimawirkungs- und Risikoanalyse (KWRA) 2021 für Deutschland, Kurzzusammenfassung, S. 1.
8) Klimawirkungs- und Risikoanalyse (KWRA) 2021 für Deutschland, Kurzzusammenfassung, S. 1.
9) Klimawirkungs- und Risikoanalyse (KWRA) 2021 für Deutschland, Kurzzusammenfassung, S. 1.
10) Klimawirkungs- und Risikoanalyse (KWRA) 2021 für Deutschland, Kurzzusammenfassung, S. 1.

Bedarf nach Klimaanpassung auslösen.[11] Dementsprechend vielgestaltig ist auch das in Betracht kommende Instrumentarium der Klimaanpassungsmaßnahmen. Teilweise stehen technologische Lösungsansätze im Vordergrund,[12] etwa beim Deichbau in Küstenregionen zur Vorsorge gegenüber steigenden Meeresspiegeln, bei Entsalzungsanlagen zur Gewinnung von Süßwasser, beim Einsatz von Bewässerungssystemen wegen Trockenheitsfolgen in der Landwirtschaft oder bei der biotechnologischen Anpassung von Saatgut an veränderte Klimabedingungen[13].

Andere Anpassungsinstrumente verfolgen naturbasierte Ansätze und sind darauf ausgerichtet, Ökosystemfunktionen zu stärken, die auf natürliche Weise nachteiligen Auswirkungen der Klimaänderungen entgegenwirken. Zu den naturbasierten Ansätzen zählen in einer Aufstellung des Umweltbundesamts „die Entsiegelung von Flächen, Schaffung von mehr Retentionsflächen (Freiräume für Gewässer), der Schutz des städtischen Grüns gegen Bebauungsdruck, mehr Bäume in den Städten und Fassaden- sowie Dachbegrünungen“,[14] aber auch „der Waldumbau zu naturnahen, standortgerechten Mischwäldern“ und „flächendeckende bodenschonende Bewirtschaftungsweisen“.[15] Technische und naturbasierte Lösungen werden auch kombiniert.

Die Vielgestaltigkeit und Kontextgebundenheit von Anpassungsbedarf und Anpassungsinstrumentarium, aber auch die Notwendigkeit zur dynamischen Fortentwicklung, verleiht dem Recht der Klimaanpassung ein eigenes Gepräge.[16] Es gibt keine übergreifende, arithmetisch darstellbare Zielgröße wie das „2 Grad, besser 1,5 Grad-Ziel“ bei der Einhegung der Treibhausgasemissionsminderungen.[17] Der großflächige Einsatz bestimmter Instrumententypen, wie etwa bei der Budgetierung von Treibhausgas-Emissionsvolumina und der Einrichtung von Emissionshandelssystemen im Bereich der Mitigation, ist nicht sachgerecht.[18] Für die zuständigen hoheitlichen Stellen kommt es bei der Ausgestaltung des Anpassungsinstrumentariums entscheidend auf die Besonderheiten einzelner Sektoren und konkrete regionale oder lokale Gegebenheiten an.[19] Deshalb sind bei raumbezogenen Maßnahmen der Klimaanpassung planerische Instrumente von besonderer Bedeutung.[20] Auf den verschiedenen Ebenen des Rechts wird jeweils die abstrakte Festlegung von Anpassungszielen mit der Verpflichtung zur nachfolgenden Prüfung spezifischer Klimawandelfolgen und zur Maßnahmenplanung verbunden.[21]

Von grundlegender Bedeutung für die Anpassungsplanung ist die Festlegung von bestimmten Szenarien des Klimawandels, auf die hin die Anpassungsmaßnahmen im jeweiligen Planungshorizont zu entwickeln sind.[22] Die Festlegung von Anpassungsszenarien stützt sich regelmäßig auf klimawissenschaftliche Expertise, die auch ihrerseits regelmäßig in Szenarien zur Dimension und Geschwindigkeit künftiger Klimaveränderungen[23] arbeitet. So zeigt das IPCC für die Klimaentwicklung im 21. Jahrhundert eine Spannbreite von möglichen globalen Temperaturerhöhungen auf, die zwischen knapp 2 Grad bei erfolgreicher Implementation der Klimaschutzziele des Pariser Abkommens und über 5 Grad in einem worst-case-Szenario liegt.[24] Entsprechende Spannbreiten – mit der Folge höchst unterschiedlicher Klimawandelfolgen und Anpassungsbedarfe – bestehen auch für die Abschätzung der Temperaturentwicklung einzelner Kontinente oder Staaten. Jede Anpassungsplanung beruht mithin idealtypisch auf der normativen Auswahl eines spezifischen Anpassungsszenarios innerhalb eines Spektrums möglicher Klimaentwicklungen. Das umweltrechtliche Vorsorgeprinzip drängt dabei darauf, bei der Festlegung von Anpassungsszenarien Freiräume für den Fall von unvorhergesehenen Verschlechterungen in der Klimaentwicklung einzustellen.[25]

### 3. Klimaanpassung als Gegenstand des Pariser Abkommens

Auf völkerrechtlicher Ebene hat die Klimaanpassung in der Rechtsentwicklung seit der Verabschiedung der Klimarahmenkonvention 1992[26] bis heute deutlich an Bedeutung gewonnen.[27] Das Pariser Abkommen (PA)[28] nennt die Anpassungsdimension des Klimaschutzrechts an zentraler Stelle. Art. 2 Abs. 1 b) PA legt als Ziel des Abkommens fest, dass „die Fähigkeit zur Anpassung an die nachteiligen Auswirkungen der Klimaänderungen erhöht und die Widerstandsfähigkeit gegenüber Klimaänderungen sowie eine hinsichtlich der Treibhausgase emissionsarme Entwicklung so gefördert wird, dass die Nahrungsmittelerzeugung nicht bedroht wird“. Diese Zielbestimmung tritt gleichrangig zum Ziel des Art. 2 Abs. 1 a) PA hinzu, die Erderwärmung gegenüber vorindustrieller Zeit auf 2 Grad, besser 1,5 Grad zu begrenzen.

Art. 7 PA konkretisiert das Anpassungsziel. Art. 7 Abs. 2 PA bezeichnet die Anpassung an Klimawandelfolgen als eine weltweite gemeinsame Herausforderung mit „loka-

11) Deutsche Anpassungsstrategie an den Klimawandel, Beschluss des Bundeskabinetts v. 17. 12. 2008, Stand 12. 5. 2022, abrufbar unter https://www.bmuv.de/fileadmin/bmu-import/files/pdfs/allgemein/application/pdf/das_gesamt_bf.pdf, S. 7, 15; *Birkmann/Blätgen*, Stichwort Klimaanpassung, in: ARL – Akademie für Raumforschung und Landesplanung (Hrsg.), Handwörterbuch der Stadt- und Raumentwicklung, 2018, 1099, 1100.
12) *Birkmann/Blätgen*, Stichwort Klimaanpassung, in: ARL – Akademie für Raumforschung und Landesplanung (Hrsg.), Handwörterbuch der Stadt- und Raumentwicklung, 2018, 1099, 1103.
13) Dazu *Saab*, Leiden Journal of International Law 29 (2016), 503, 508 ff.
14) UBA, Klimaanpassung jetzt gesetzlich verankern – Vorschläge für eine wirksame Vorsorge vor Klimarisiken, Stand 19. 5. 2021, abrufbar unter https://www.umweltbundesamt.de/sites/default/files/medien/2546/dokumente/uba_vorschlaege_zur_klimaanpassung.pdf.
15) UBA, Klimaanpassung jetzt gesetzlich verankern – Vorschläge für eine wirksame Vorsorge vor Klimarisiken, Stand 19. 5. 2022, abrufbar unter https://www.umweltbundesamt.de/sites/default/files/medien/2546/dokumente/uba_vorschlaege_zur_klimaanpassung.pdf.
16) Vgl. *Kment*, JZ 2010, 62, 70.
17) Vgl. *Mayer*, Virginia Environmental Law Journal 39 (2021), 141, 164 f.
18) *Reese*, in: Marx (Hrsg.), Klimaanpassung in Forschung und Politik, 2017, 73, 74.
19) *Richardson*, in: Farber/Peeters (Hrsg.), Climate Change Law, 2016, 80, 81 f.
20) *Birkmann/Blätgen*, Stichwort Klimaanpassung, in: ARL – Akademie für Raumforschung und Landesplanung (Hrsg.), Handwörterbuch der Stadt- und Raumentwicklung, 2018, 1099, 1100 ff.; *Groth/Bender/Groth*, ZfU 2021, 385, 390.
21) *Reese*, in: Marx (Hrsg.), Klimaanpassung in Forschung und Politik, 2017, 73, 76 ff.; *Kloepfer*, EurUP 2018, 34, 37.
22) *Farber*, Journal of Environmental Law 23 (2011), 359, 373 ff.
23) Dazu Komm., Mitt. v. 24. 2. 2021, Ein klimaresilientes Europa aufbauen – die neue EU-Strategie für die Anpassung an den Klimawandel, COM(2021) 82 final, S. 5 ff.
24) IPCC, Climate Change 2021, The Physical Science Basis, Summary for Policymakers, Stand 22. 11. 2021, abrufbar unter https://www.ipcc.ch/report/ar6/wg1/#SPM, S. 13.
25) Vgl. die Empfehlung des UBA, aus Vorsorgegründen für Deutschland von einem „starken Klimawandel“ bis zur Mitte des 21. Jahrhunderts von etwa 3 Grad auszugehen, UBA, Klimaanpassung jetzt gesetzlich verankern – Vorschläge für eine wirksame Vorsorge vor Klimarisiken, Stand 14. 5. 2022, abrufbar unter https://www.umweltbundesamt.de/sites/default/files/medien/2546/dokumente/uba_vorschlaege_zur_klimaanpassung.pdf.
26) United Nations Framework Convention on Climate Change (UNFCCC) v. 9. 5. 1992, abgedruckt mit Zustimmungsgesetz v. 13. 9. 1993, BGBl. II S. 1784.
27) *Mayer*, Virginia Environmental Law Journal 39 (2021), 141, 147.
28) Pariser Abkommen v. 12. 12. 2015, abgedruckt mit Zustimmungsgesetz v. 28. 9. 2016, BGBl. II S. 1082.

len, subnationalen, nationalen, regionalen und internationalen Dimensionen“, bei der es um den „Schutz der Menschen, der Existenzgrundlagen und der Ökosysteme“ gehe. Zugleich trägt Art. 7 Abs. 2 PA aber auch der besonderen Betroffenheit bestimmter Vertragsparteien Rechnung und verpflichtet zur Berücksichtigung der „vordringlichen und unmittelbaren Bedürfnisse der für die nachteiligen Auswirkungen der Klimaänderungen besonders anfälligen Vertragsparteien, die Entwicklungsländer sind“. Art. 7 Abs. 4 PA betont die Wechselbezüglichkeit zwischen der Erfolgsbilanz hinsichtlich Erreichung der Emissionsminderungsziele und dem Anpassungsbedarf: Ein „höheres Minderungsniveau [kann] die Notwendigkeit zusätzlicher Anpassungsbemühungen verringern“. Nach Art. 7 Abs. 7 PA sollen die Vertragsparteien verstärkt in Anpassungsfragen kooperieren, unter anderem in der Form gegenseitiger technischer Unterstützung und Beratung.[29] Die Mitgliedstaaten werden in Art. 7 Abs. 9–12 PA verpflichtet, nationale Pläne zur Anpassung und zur Durchführung von Maßnahmen sowie konkretisierende Anpassungsmitteilungen vorzulegen, wobei die Vorlagepflicht durch den Begriff „gegebenenfalls“ (englische Sprachfassung: „as appropriate“) in Art. 7 Abs. 9 PA relativiert wird.[30] Art. 9 PA sieht vor, dass die entwickelten Länder unter den Vertragsparteien finanzielle Mittel für Maßnahmen der Emissionsreduktion, aber auch für Anpassungsmaßnahmen zugunsten von Vertragsparteien, die Entwicklungsländer sind, bereitstellen.[31]

In dem als „Glasgow Climate Pact“ verabschiedeten Beschluss der 26. Vertragsstaatenkonferenz der Klimarahmenkonvention von Glasgow im November 2021 (COP26)[32] hat die Klimaanpassung eine hervorgehobene Bedeutung.[33] Der Beschluss betont die Dringlichkeit erhöhter internationaler Anstrengungen im Bereich der Klimaanpassung durch Klimafinanzierung, Kapazitätsaufbau und Technologietransfers.[34] Für die Finanzierung von Anpassungsmaßnahmen sieht der Beschluss die Stärkung zweier Fonds auf UN-Ebene vor, nämlich des Adaptation Fund und des Least Developed Countries Fund.[35] Zur Vorbereitung einer weiteren Konkretisierung des Anpassungsziels des PA auf der kommenden Weltklimakonferenz in Ägypten (COP27) wurde das „Glasgow–Sharm el-Sheikh work programme on the global goal on adaptation“ etabliert.[36]

## 4. Klimaanpassung im EU-Recht

Die Europäische Union möchte bis 2050 das Ziel der „klimaresilienten Union“ und der vollständigen Anpassung an die unausweichlichen Auswirkungen des Klimawandels erreichen.[37]

### *4.1 Europäisches Klimagesetz*

Die auch als „Europäisches Klimagesetz“ bezeichnete VO 2021/1119/EU[38] regelt die Anpassung an den Klimawandel in Art. 5. Die Vorschrift verpflichtet in Art. 5 Abs. 1 die Organe der Union und die Mitgliedstaaten dazu „für kontinuierliche Fortschritte bei der Verbesserung der Anpassungsfähigkeit, der Stärkung der Widerstandsfähigkeit und der Verringerung der Anfälligkeit gegenüber Klimaänderungen“ zu sorgen. Ausdrücklich nimmt Art. 5 Abs. 1 Europäisches Klimagesetz dabei auf die korrespondierende Vorschrift des Art. 7 PA Bezug.[39] Art. 5 Abs. 2 Europäisches Klimagesetz festigt die für die EU als Mitglied des Pariser Abkommens bereits bestehende Pflicht (s. oben 3.) zur Annahme einer Strategie zur Anpassung an den Klimawandel. Art. 5 Abs. 4 Europäisches Klimagesetz verpflichtet die Mitgliedstaaten zur Annahme und Umsetzung nationaler Anpassungsstrategien und Anpassungspläne, die wiederum mit der EU-Anpassungsstrategie abzustimmen sind.[40] Die nationalen Anpassungsstrategien unterliegen gem. Art. 7 Abs. 1 b) Europäisches Klimagesetz der Kontrolle und Bewertung der Kommission, wobei die erstmalige Kontrolle bis zum 30. September 2023 und danach alle fünf Jahre vorgesehen ist. Die Kommission kann zu den nationalen Anpassungsstrategien gem. Art. 7 Abs. 2 Europäisches Klimagesetz Empfehlungen aussprechen, die öffentlich zugänglich sind.[41] Zudem verpflichtet Art. 5 Abs. 5 Europäisches Klimagesetz die Kommission, bis zum 30. 7. 2022 Leitlinien zur Ermittlung, Einstufung und aufsichtsrechtlichen Bewältigung von Klimarisiken bei Projekten und Programmen zu erlassen.[42]

### *4.2 Spezifische Politikbereiche*

Zusätzlich zu den übergreifenden Vorgaben des Europäischen Klimagesetzes enthält das Unionsrecht in zahlreichen weiteren Rechtsakten Vorgaben für die Klimaanpassung in spezifischen Politikbereichen. Beispielsweise spielen Aspekte der Klimaanpassung inzwischen im Recht der Umweltverträglichkeitsprüfung eine große Rolle. Die Änderungsrichtlinie 2014/52/EU[43] zur UVP-Richtlinie 2011/92/EU hat die Bedeutung von Anpassungsbelangen erheblich gestärkt. Heute sind Folgen des Klimawandels ein zentraler Aspekt bei der Ermittlung der Umweltauswirkungen der von der UVP-Richtlinie erfassten öffentlichen und privaten Projekten, zu denen insbesondere umweltrelevante Industrieanlagen und Verkehrsinfrastrukturen gehören. Das Ziel ist die Sicherung der Klimaverträglichkeit bzw. in Übernahme des englischsprachigen Grundbegriffs das „climate proofing“ von Infrastrukturprojekten.[44] Gem. Erwägungsgrund (13) der Richt-

29) Dazu *Rajamani*, Journal of Environmental Law 28 (2016), 337, 344 ff.
30) Dazu *Rajamani*, Journal of Environmental Law 28 (2016), 337, 352 f.
31) Kritisch wegen fehlender Effektivität der Finanzierungsverpflichtungen *Mayer*, Virginia Environmental Law Journal 39 (2021), 141, 159 f.
32) Decision -/CP.26 Glasgow Climate Pact, Stand 18.5.2022, abrufbar unter https://unfccc.int/sites/default/files/resource/cop26_auv_2f_cover_decision.pdf.
33) Vgl. die Darstellung, Stand 18.5.2022, abrufbar unter https://www4.unfccc.int/sites/NWPStaging/News/Pages/Adaptation-at-the-forefront-of-COP-26-outcomes-in-Glasgow.aspx.
34) Decision -/CP.26 Glasgow Climate Pact, Nr. 6, Stand 18.5.2022, abrufbar unter https://unfccc.int/sites/default/files/resource/cop26_auv_2f_cover_decision.pdf.
35) Decision -/CP.26 Glasgow Climate Pact, Nr. 13, Stand 18.5.2022, abrufbar unter https://unfccc.int/sites/default/files/resource/cop26_auv_2f_cover_decision.pdf.
36) Decision -/CMA.3 Glasgow–Sharm el-Sheikh work programme on the global goal on adaptation; Stand 18.5.2022, abrufbar unter https://unfccc.int/sites/default/files/resource/cma3_auv_4ac_Global_Goal.pdf.
37) Komm., Mitt. v. 24.2.2021, Ein klimaresilientes Europa aufbauen – die neue EU-Strategie für die Anpassung an den Klimawandel, COM(2021) 82 final, S. 3.
38) Verordnung (EU) 2021/1119 des Europäischen Parlaments und des Rates vom 30.6.2021 zur Schaffung des Rahmens für die Verwirklichung der Klimaneutralität und zur Änderung der Verordnungen (EG) Nr. 401/2009 und (EU) 2018/1999 („Europäisches Klimagesetz“), ABl. 2021 L 243, S. 1.
39) *Frenz*, in: Frenz (Hrsg.), Klimaschutzrecht – Gesamtkommentar, 2. Aufl. 2022, Art. 5 EU-KG, Rdnr. 3 ff.
40) *Frenz*, in: Frenz (Hrsg.), Klimaschutzrecht – Gesamtkommentar, 2. Aufl. 2022, Art. 5 EU-KG, Rdnr. 26 ff.
41) *Schlacke/Köster/Thierjung*, EuZW 2021, 620, 622 f.
42) *Schlacke/Köster/Thierjung*, EuZW 2021, 620, 622.
43) Richtlinie 2014/52/EU des Europäischen Parlaments und des Rates vom 16. April 2014 zur Änderung der Richtlinie 2011/92/EU über die Umweltverträglichkeitsprüfung bei bestimmten öffentlichen und privaten Projekten, ABl. 2014 L 124, S. 1.
44) Vgl. die Bekanntmachung der Kommission, Technische Leitlinien für die Sicherung der Klimaverträglichkeit von Infrastrukturen im Zeitraum 2021–2027, ABl. 2021 C 373, S. 1; die Bezeichnung in der englischen Sprachfassung lautet „Technical guidance on the climate proofing of infrastructure in the period 2021–2027“.

linie 2014/52/EU ist es wegen der durch den Klimawandel verursachten Umweltschäden und deren potenziellen negativer ökologischen Folgen angezeigt, neben der Untersuchung der Klimawirkungen von UVP-pflichtigen Projekten durch deren Treibhausgasemissionen auch „ihre Anfälligkeit in Bezug auf den Klimawandel zu bewerten". Gem. Art. 5 Abs. 1 i.V.m. Anhang IV, Nr. 5 f) der UVP-Richtlinie i.d.F. der Änderungsrichtlinie 2014/52/EU ist eine Beschreibung der „Anfälligkeit des Projekts in Bezug auf den Klimawandel" ein verpflichtender Bestandteil des UVP-Berichts und demgemäß in Zusammenschau mit den zur Anpassung an Klimawandelfolgen getroffenen Maßnahmen in der Abwägung hinsichtlich der Projektzulassung zu berücksichtigen.[45] Auch bei der UVP-Vorprüfung sind Klimawandelfolgen relevant. Gem. Art. 4 Abs. 3 i.V.m. Anhang III, Nr. 1 f) der UVP-Richtlinie i.d.F. der Änderungsrichtlinie 2014/52/EU ist das zu prüfende Projekt auch im Hinblick auf „Risiken schwerer Unfälle und/oder von Katastrophen, die für das betroffene Projekt relevant sind, einschließlich solcher, die wissenschaftlichen Erkenntnissen zufolge durch den Klimawandel bedingt sind" zu beurteilen.

Im Bereich der Infrastrukturförderung durch die EU verknüpft etwa die Dachverordnung für Fonds mit geteilter Mittelverwaltung VO 2021/1060/EU die Förderung von mittel- und langfristigen Infrastrukturinvestitionen mit dem Gebot der Sicherung der Klimaverträglichkeit, das auf Verfahrensebene verlangt zu verhindern, dass Infrastrukturen durch potenzielle langfristige Auswirkungen des Klimawandels gefährdet werden.[46] Im Rahmen der durch VO 2021/241/EU[47] im Kontext der Bewältigung der COVID 19-Pandemie errichteten Aufbau- und Resilienzfazilität[48] ist die Förderung von Aufbau- und Resilienzplänen der Mitgliedstaaten gem. Erwägungsgrund (23) und Art. 16 Abs. 2 lit. b) i) VO 2021/241/EU auch an deren Ausrichtung auf den Klimaschutz geknüpft. Der konkretisierende Anhang VI zur VO stuft „Maßnahmen zur Anpassung an den Klimawandel und Vorbeugung und Bewältigung klimabezogener Risiken" ausdrücklich als förderfähig ein und nimmt dabei auf Hochwasser, Brände, Stürme und Dürren Bezug.[49] Als förderfähige Maßnahmen werden beispielhaft Sensibilisierungsmaßnahmen, Einrichtungen im Bereich Katastrophenschutz und -bewältigung, Infrastrukturanlagen sowie ökosystembasierte Ansätze genannt.[50]

## 5. Bundesrecht

### *5.1. Deutsche Anpassungsstrategie an den Klimawandel 2008 und Entwicklung im Fachrecht*

Im Bundesrecht gibt es bislang keine übergreifende gesetzliche Grundlage für das Recht der Klimaanpassung. Vorschläge zur Regelung im Bundes-Klimaschutzgesetz scheiterten (s. sogleich 5.2). Allerdings sind in zahlreichen Fachgesetzen sektorale Regelungen zur Klimaanpassung entstanden. Mit der Novelle des Raumordnungsgesetzes 2008[51] erfolgte eine Erweiterung der Ziele der Raumordnung um die Beachtung von Folgen des Klimawandels.[52] §2 Abs. 2 Nr. 6 Raumordnungsgesetz statuiert das Ziel der Raumordnung, den „räumlichen Erfordernissen des Klimaschutzes … Rechnung zu tragen, sowohl durch Maßnahmen, die dem Klimawandel entgegenwirken, als auch durch solche, die der Anpassung an den Klimawandel dienen".[53] Im UVP-Gesetz erfolgte die Umsetzung der Vorgaben der auf die Klimaanpassung bezogenen UVP-Änderungsrichtlinie 2014/52/EU. Dazu wurden Aspekte der Klimaanpassung in die Kriterien für die Vorprüfung und den UVP-Bericht aufgenommen.[54] Gem. §7 Abs. 1 i.V.m. Anl. 3 Nr. 1.6 UVPG ist die UVP-Vorprüfung auch zu beziehen auf „Risiken von Störfällen, Unfällen und Katastrophen, die für das Vorhaben von Bedeutung sind, einschließlich der Störfälle, Unfälle und Katastrophen, die wissenschaftlichen Erkenntnissen zufolge durch den Klimawandel bedingt sind." Gem. §16 i.V.m. Anl. 4, Nr. 4, c), hh) UVPG muss der UVP-Bericht „die Anfälligkeit des Vorhabens gegenüber den Folgen des Klimawandels (zum Beispiel durch erhöhte Hochwassergefahr am Standort)" beschreiben. Im WHG wurde 2009[55] in §6 Abs. 1 Nr. 5 WHG die Vorbeugung vor möglichen Folgen des Klimawandels als allgemeiner Grundsatz der Gewässerbewirtschaftung ergänzt.[56] Zugleich wurden im Abschnitt zum Hochwasserschutz aufgrund europarechtlicher Vorgaben §73 Abs. 6 S. 2 WHG und §75 Abs. 6 S. 3 WHG neu eingefügt, die verlangen, dass bei Risikobewertung, Bestimmung der Risikogebiete und bei Risikomanagementplänen auch die voraussichtlichen Auswirkungen des Klimawandels auf das Hochwasserrisiko zu berücksichtigen sind.[57] Im BauGB wurden im allgemeinen und im besonderen Städtebaurecht verschiedene Vorgaben zur Klimaanpassung eingefügt (dazu noch unten 5.2).[58]

Die wichtigste sektorübergreifende Regelung zur Klimaanpassung auf Bundesebene ist bislang die „Deutsche Anpassungsstrategie an den Klimawandel", die erstmals 2008[59] als einfacher Beschluss der Bundesregierung verabschiedet worden ist.[60] Die Deutsche Anpassungsstrategie wurde als mittelfristiger Prozess zur schrittweisen Benennung von Handlungsbedarfen konzipiert[61] und strebt „sektor- und bereichsübergreifende, integrale Ansätze" an, um Nutzungs- und Zielkonflikten infolge vom Klimaänderungen und Folgen vorzubeugen.[62] Im Jahr 2011 verabschie-

45) Vgl. *Balla/Peters*, NuR 2015, 297, 302.
46) Art. 2 Nr. 37 und Art. 67 Abs. 3 j) der Verordnung (EU) 2021/1060 des Europäischen Parlaments und des Rates vom 24.6.2021 mit gemeinsamen Bestimmungen für den Europäischen Fonds für regionale Entwicklung, den Europäischen Sozialfonds Plus, den Kohäsionsfonds, den Fonds für einen gerechten Übergang und den Europäischen Meeres-, Fischerei- und Aquakulturfonds sowie mit Haushaltsvorschriften für diese Fonds und für den Asyl-, Migrations- und Integrationsfonds, den Fonds für die innere Sicherheit und das Instrument für finanzielle Hilfe im Bereich Grenzverwaltung und Visumpolitik, ABl. 2021 L 231, S. 159.
47) Verordnung (EU) 2021/241 des Europäischen Parlaments und des Rates vom 12.1.2021 zur Einrichtung der Aufbau- und Resilienzfazilität, ABl. 2021 L 57, S. 17.
48) Zu den kompetenzrechtlichen Grundlagen *De Witte*, Common Market Law Review 58 (2021), 635, 652ff.
49) VO 2021/241/EU, Anhang VI, Methodik zur Verfolgung von Klimamaßnahmen, Ziff. 35, 36 und 37.
50) VO 2021/241/EU, Anhang VI, Methodik zur Verfolgung von Klimamaßnahmen, Ziff. 35, 36 und 37.
51) Gesetz zur Neufassung des Raumordnungsgesetzes und zur Änderung anderer Vorschriften v. 22.12.2008, BGBl. I S. 2986.
52) Dazu *Bubeck/Klimmer/Albrecht*, NuR 2016, 297, 298ff.; *Fischer*, Grundlagen und Grundstrukturen eines Klimawandelanpassungsrechts, 2013, S. 232ff.
53) Näher *Kment*, JZ 2010, 62, 69; *Bubeck/Klimmer/Albrecht*, NuR 2016, 297, 300f.; *Groth/Bender/Groth*, ZfU 2021, 385, 390.
54) Vgl. UVP-Änderungsgesetz v. 30.11.2016, BGBl. I S. 2753; Gesetz zur Modernisierung des Rechts der Umweltverträglichkeitsprüfung v. 20.7.2017, BGBl. I S. 2808.
55) Gesetz zur Neuregelung des Wasserrechts v. 31.7.2009, BGBl. I S. 2585.
56) Dazu *Reese*, NuR 2011, 19, 21.
57) Dazu *Köck*, ZUR 2015, 515, 517f.; *Bubeck/Klimmer/Albrecht*, NuR 2016, 297, 304.
58) Im Überblick *Groth/Bender/Groth*, ZfU 2021, 385, 391ff.; Bubeck/*Klimmer/Albrecht*, NuR 2016, 297, 301ff.
59) Deutsche Anpassungsstrategie an den Klimawandel, Beschluss des Bundeskabinetts v. 17.12.2008, Stand 12.5.2022, abrufbar unter https://www.bmuv.de/fileadmin/bmu-import/files/pdfs/allgemein/application/pdf/das_gesamt_bf.pdf.
60) *Kment*, JZ 2010, 62, 68; *Kloepfer*, EurUP 2018, 34, 39; *Fischer*, Grundlagen und Grundstrukturen eines Klimawandelanpassungsrechts, 2013, S. 80ff.
61) Deutsche Anpassungsstrategie an den Klimawandel, Beschluss des Bundeskabinetts v. 17.12.2008, S. 6.
62) Deutsche Anpassungsstrategie an den Klimawandel, Beschluss des Bundeskabinetts v. 17.12.2008, S. 7.

dete die Bundesregierung den „Aktionsplan Anpassung der deutschen Anpassungsstrategie an den Klimawandel".[63] Die Deutsche Anpassungsstrategie wurde durch Fortschrittsberichte der Bundesregierung 2015[64] und 2020[65] aktualisiert.

### *5.2 Rechtspolitische Initiativen: Klimaanpassung im Bundes-Klimaschutzgesetz und als Gemeinschaftsaufgabe im Grundgesetz*

Der Bundesrat hat im Rahmen der Novelle des Bundes-Klimaschutzgesetzes 2021 vorgeschlagen, die Klimaanpassung auch im Bundes-Klimaschutzgesetz zu verankern. Die Länderkammer unterbreitete einen Vorschlag zur Einfügung eines neuen § 3 Abs. 5 KSG zur Verankerung der Ziele Verbesserung der Anpassungsfähigkeit an den Klimawandel, Stärkung der Widerstandsfähigkeit und Verringerung der Anfälligkeit gegenüber Klimaänderungen, der jedoch ohne Erfolg blieb.[66] Auch der verfassungsrechtliche Rahmen der Klimaanpassung war bereits Gegenstand rechtspolitischer Vorschläge. So plädierte das Umweltbundesamt[67] für die Einfügung einer neuen Gemeinschaftsaufgabe „Anpassung an den Klimawandel" in das Grundgesetz. Die verfassungsrechtliche Kategorie der „Gemeinschaftsaufgaben" hat ihre zentrale Vorschrift in Art. 91a GG. Sie erfasst Sachbereiche, in denen die grundsätzliche Trennung der Verwaltungsräume von Bund und Ländern zugunsten einer ausnahmsweisen Mischverwaltung aufgehoben ist.[68] Ist ein bestimmter Sachbereich verfassungsrechtlich als Gemeinschaftsaufgabe ausgewiesen, so geht damit insbesondere eine Finanzierungsverantwortung des Bundes einher.[69] Auch der Koalitionsvertrag 2021–2025 von SPD, FDP und Bündnis 90/Die Grünen vom Dezember 2021 enthält das Ziel einer „Verankerung der gemeinsamen Finanzierung von Bund und Ländern zur Klimavorsorge und Klimaanpassung" und der Ausstattung mit „ausreichend finanziellen Mitteln", allerdings ohne ausdrückliche Bezugnahme auf das etwaige Erfordernis einer Verfassungsänderung.[70]

### *5.3 Das Projekt eines Klimaanpassungsgesetzes des Bundes*

Der Koalitionsvertrag 2021–2025 von SPD, FDP und Bündnis 90/Die Grünen sieht vor, auf Bundesebene ein „Klimaanpassungsgesetz" zu schaffen.[71] Dieses neu zu erlassende Gesetz soll für den Bund den rechtlichen Rahmen schaffen, um „gemeinsam mit den Ländern eine nationale Klimaanpassungsstrategie mit messbaren Zielen etwa in den Handlungsfeldern Hitzevorsorge, Gesundheits- und Allergieprävention und Wasserinfrastruktur umzusetzen und rechtzeitig nachsteuern zu können".[72] Kompetenzrechtlich wirft das Projekt eines Klimaanpassungsgesetzes des Bundes Klärungsbedarf auf. Maßgeblich kann hier anders als beim Bundes-Klimaschutzgesetz[73] nicht die übergreifende Bezugnahme auf die konkurrierende Gesetzgebungskompetenz für die Luftreinhaltung sein, sondern es wird je nach Zuschnitt der Regelungsgegenstände des Gesetzes auf verschiedene Bundeskompetenzen ankommen, so etwa auf die Förderung der land- und forstwirtschaftlichen Erzeugung und die Sicherung der Ernährung (Art. 74 Abs. 1 Nr. 17 GG), den Naturschutz und die Landschaftspflege (Art. 74 Abs. 1 Nr. 29 GG), die Bodenverteilung (Art. 74 Abs. 1 Nr. 30 GG), die Raumordnung (Art. 74 Abs. 1 Nr. 31 GG) und den Wasserhaushalt (Art. 74 Abs. 1 Nr. 32 GG).[74] In Ergänzung zum langfristig angelegten Klimaanpassungsgesetz des Bundes sieht der Koalitionsvertrag auch die kurzfristige Aufstellung eines Sofortprogramms vor.[75]

Als zukünftige Handlungsschwerpunkte von Recht und Politik der Klimaanpassung auf Bundesebene nennt der Koalitionsvertrag zum einen die Anpassung und Neuschaffung von bundesweit maßgeblichen Regelwerken und Standards im Lichte des Klimawandels,[76] zum anderen die finanzielle Unterstützung von Klimaanpassungsmaßnahmen der Länder und Kommunen, aber auch von Privatpersonen, etwa bei der Hochwasser- und Starkregenvorsorge. Gerade der Handlungsschwerpunkt des Bundes in der föderalen Allokation von Fördermitteln lässt erwarten, dass sich das Klimaanpassungsrecht in besonders starkem Maße als Kooperationsrecht im Zusammenwirken von Bund, Ländern und Gemeinden entwickeln wird. Auf dieser Entwicklungslinie liegt auch der oben genannte Vorschlag für eine Verfassungsänderung durch Ergänzung des Art. 91a GG um eine Gemeinschaftsaufgabe „Klimaanpassung" (s. oben 5.2.).

Die föderal-kooperative Perspektive ist von den tatsächlichen Herausforderungen des Klimawandels her geboten, da die konkreten Klimaanpassungsbedarfe über die Bundesländer und Naturräume hinweg bereits heute stark unterschiedlich ausfallen und auch perspektivisch eine divergierende Entwicklung mit Ausprägung regionaler Besonderheiten und Extreme zu erwarten ist.[77] Zu achten ist bei der rechtlichen Ausgestaltung auf die Abstimmung mit dem EU-Recht, insbes. auf die Verpflichtung zur Erstellung und zur regelmäßigen Aktualisierung von nationalen Anpassungsstrategien gem. Art. 5 Abs. 4 EU-Klimagesetz (vgl. oben 4.1.).

63) Aktionsplan Anpassung der Deutschen Anpassungsstrategie an den Klimawandel, Beschluss des Bundeskabinetts v. 31. 8. 2011, Stand 17. 5. 2022, abrufbar unter https://www.bmuv.de/fileadmin/bmu-import/files/pdfs/allgemein/application/pdf/aktionsplan_anpassung_klimawandel_bf.pdf.

64) Fortschrittsbericht der Bundesregierung zur Deutschen Anpassungsstrategie an den Klimawandel, Stand 12. 5. 2022, abrufbar unter https://www.bmuv.de/fileadmin/Daten_BMU/Download_PDF/Klimaschutz/klimawandel_das_fortschrittsbericht_bf.pdf.

65) Zweiter Fortschrittsbericht der Bundesregierung zur Deutschen Anpassungsstrategie, 2020, Stand 12. 5. 2022, abrufbar unter https://www.bmuv.de/fileadmin/Daten_BMU/Download_PDF/Klimaschutz/klimawandel_das_2_fortschrittsbericht_bf.pdf.

66) Vgl. BT-Drs. 19/30230, Anl. 3, S. 31 und 37.

67) UBA, Klimaanpassung jetzt gesetzlich verankern – Vorschläge für eine wirksame Vorsorge vor Klimarisiken, Stand 22. 7. 2021, abrufbar unter https://www.umweltbundesamt.de/sites/default/files/medien/2546/dokumente/uba_vorschlaege_zur_klimaanpassung.pdf.

68) *Heun*, in: Dreier (Hrsg.), GG, Bd. 3, 3. Aufl. 2018, Art. 91a, Rdnr. 7.

69) *Heun*, in: Dreier (Hrsg.), GG, Bd. 3, 3. Aufl. 2018, Art. 91a, Rdnr. 7 ff.

70) Koalitionsvertrag 2021–2025 von SPD, FDP und Bündnis 90/Die Grünen, Stand 17. 5. 2022, abrufbar unter https://www.bundesregierung.de/breg-de/service/gesetzesvorhaben/koalitionsvertrag-2021-1990800, S. 40.

71) Koalitionsvertrag 2021–2025 von SPD, FDP und Bündnis 90/Die Grünen, abrufbar unter https://www.bundesregierung.de/breg-de/service/gesetzesvorhaben/koalitionsvertrag-2021-1990800, S. 40.

72) Koalitionsvertrag 2021–2025 von SPD, FDP und Bündnis 90/Die Grünen, Stand 17. 5. 2022, abrufbar unter https://www.bundesregierung.de/breg-de/service/gesetzesvorhaben/koalitionsvertrag-2021-1990800, S. 40.

73) Näher *Saurer*, in: Frenz (Hrsg.), Klimaschutzrecht – Gesamtkommentar, 2. Aufl. 2022, Einl., Rdnr. 46 ff.

74) Vgl. *Köck/Kohlrausch*, ZUR 2021, 610, 611.

75) Koalitionsvertrag 2021–2025 von SPD, FDP und Bündnis 90/Die Grünen, Stand 17. 5. 2022, abrufbar unter https://www.bundesregierung.de/breg-de/service/gesetzesvorhaben/koalitionsvertrag-2021-1990800, S. 40.

76) Koalitionsvertrag 2021–2025 von SPD, FDP und Bündnis 90/Die Grünen, Stand 17. 5. 2022, abrufbar unter https://www.bundesregierung.de/breg-de/service/gesetzesvorhaben/koalitionsvertrag-2021-1990800, S. 40; konkret nennt der Koalitionsvertrag das Ziel der Schaffung von „bundeseinheitliche[n] Standards für die Bewertung von Hochwasser- und Starkregenrisiken und die Erstellung und Veröffentlichung von Gefahren- und Risikokarten".

77) Vgl. zu regionalen Besonderheiten und Extremen die Beiträge u. a. zu Hitze, Niederschlag, Wind, Meeresspiegel, Hochwasser und Dürre in *Brasseur/Jacob/Schuck-Zöller* (Hrsg.), Klimawandel in Deutschland, 2017, S. 47–126.

## 6. Bundesländer und Kommunen

Die gesetzliche Ausgestaltung des Klimaanpassungsrechts ist in den deutschen Bundesländern auf der sektorübergreifenden Ebene schon deutlich weiter entwickelt als auf Bundesebene.

### *6.1 Landesgesetze mit Vorgaben zur Klimaanpassung*

Zahlreiche Bundesländer haben gesetzliche Grundlagen für Ziele und konkretisierende Maßgaben der Klimaanpassung geschaffen. Dazu wurde die Aufgabe der Klimaanpassung zumeist als weitere Dimension des Klimaschutzrechts neben den Vorgaben zur Minderung von Treibhausgas-Emissionsminderungen in die einschlägigen Landesgesetze aufgenommen,[78] so etwa in § 4a Klimaschutzgesetz Baden-Württemberg,[79] § 15 Berliner Klimaschutz- und Energiewendegesetz,[80] § 10 Energiewende- und Klimaschutzgesetz Schleswig-Holstein,[81] § 2 Abs. 3 Nr. 3 und § 5 Hamburgisches Klimaschutzgesetz[82] und Art. 5 Bayerisches Klimaschutzgesetz.[83] Teilweise kommt die Erfassung der Anpassungsdimension bereits im Gesetzestitel zum Ausdruck, so im Thüringer Gesetz zum Klimaschutz und zur Anpassung an die Folgen des Klimawandels.[84] Das Land Nordrhein-Westfalen hat für Klimaschutz und Klimaanpassung gesonderte Landesgesetze geschaffen. Neben dem Landes-Klimaschutzgesetz Nordrhein-Westfalen[85] besteht seit 2021 ein eigenständiges Klimaanpassungsgesetz Nordrhein-Westfalen.[86]

Die Anpassungsziele der Landesgesetze sind teilweise offen als Anpassung an die Folgen des Klimawandels formuliert wie in § 5 Abs. 1 HmbKlG oder § 1 EWKG SH, teilweise aber auch ausdrücklich auf die Begrenzung von (negativen) Auswirkungen des Klimawandels ausgerichtet wie in § 3 Abs. 1 KlAnG NRW.[87] Die Begründung des Gesetzentwurfs zum KlAnG NRW konkretisiert die Klimaanpassungsziele mit einer beispielhaft zu verstehenden positiven Umschreibung, die auf „Erhöhung der Widerstandsfähigkeit von Siedlungs- und Infrastrukturen, die Erhaltung der Stabilität und Leistungsfähigkeit von Naturhaushalt und Ökosystemen oder die Einstellung der Forst- und Landwirtschaft auf veränderte Klimabedingungen" abstellt.[88]

Die Landes-Klimaschutzgesetze verbinden die Anpassungsziele regelmäßig mit der Verpflichtung der jeweiligen Landesregierung zur Anpassungsplanung bzw. zur Verabschiedung einer Anpassungsstrategie und zur späteren Fortschreibung.[89] Entsprechende Vorgaben existieren etwa in § 4a S. 2 KSG Baden-Württemberg,[90] in Art. 5 Abs. 1 Nr. 2 BayKlimaG und in § 15 EWKG Schleswig-Holstein. Gem. § 11 Abs. 1 S. 1 ThürKlimaG beschließt die Landesregierung ein „Integriertes Maßnahmenprogramm zur Anpassung an die Folgen des Klimawandels in Thüringen". § 11 Abs. 1 S. 2 ThürKlimaG gibt zum Inhalt vor, dass dieses Maßnahmenprogramm „unter der Berücksichtigung der Aspekte der Finanzierbarkeit und Wirtschaftlichkeit erstellt [wird] und … sektorspezifische Strategien und Maßnahmen zur Begrenzung der negativen Folgen des Klimawandels [enthält]". § 6 Abs. 1 HmbGKlimG ordnet an, dass die „der Anpassung an den Klimawandel dienenden Maßnahmen" gemeinsam mit den Sektorzielen und Maßnahmen für die Emissionsminderung durch den Senat im Hamburger Klimaplan festgelegt werden, der alle vier Jahre fortzuschreiben ist.[91]

Mehrere Bundesländer haben spezifische gesetzliche Vorgaben für die Erstellung und Aktualisierung der Anpassungsplanung festgelegt, die unterschiedliche Akzente setzen.[92] Gem. § 11 Abs. 2 ThürKlimaG ist das Integrierte Maßnahmenprogramm zur Anpassung an die Folgen des Klimawandels vor der Beschlussfassung dem Landtag mit der Gelegenheit zur Stellungnahme zuzuleiten. § 4 Abs. 3 EWG Bln sieht sogar einen Beschluss des Abgeordnetenhauses über das Energie- und Klimaschutzprogramm Berlin vor.[93] § 4a S. 2 KSG BW verpflichtet die Landesregierung im Vorfeld der Verabschiedung der Anpassungsstrategie zur Anhörung von Verbänden und Vereinigungen. Weiterhin hat die Landesregierung gem. § 10 Abs. 1 KSG BW bei der Festlegung der Anpassungsstrategie und bei der Weiterentwicklung der Anpassungsmaßnahmen den Rat des Klima-Sachverständigenrats des Landes einzuholen. Auch § 11 KlAnG NRW und § 14 ThürKlimaG sehen die Einrichtung von Beiräten zur beratenden Begleitung der Klimaanpassungspolitik des Landes vor.

Zudem ordnen die Landesgesetze Monitoringprozesse für die Klimaanpassungsstrategien und die Umsetzung der Anpassungsmaßnahmen an, so in § 16 EWG Bln, § 9 KlAnG NRW oder § 13 ThürKlimaG.[94] Die Monitoringberichte bilden die Grundlage für die ebenfalls regelmäßig vorgesehene Pflicht der Landesregierungen zur Fortschreibung der Anpassungspläne, so etwa gem. § 4a S. 2 i. V. m. § 9 Abs. 2 S. 1 Nr. 3 KSG BW oder gem. § 13 ThürKlimaG innerhalb eines Zeitraums von fünf Jahren.

### *6.2 Klimaanpassung in den Kommunen*

Die kommunale Ebene spielt im Mehr-Ebenen-System des Rechts der Klimaanpassung eine wichtige Rolle. So betont etwa die EU-Kommission in ihrer Mitteilung zur Klimaanpassung vom 24. 2. 2021 die grundlegende Bedeutung der lokalen Ebene für die Klimaanpassung und die dementsprechende Zielsetzung der EU-Unterstützung zur „Steigerung der lokalen Resilienz".[95] Als konkrete Ressourcen für die Förderung kommunaler Klimaanpassungsmaßnahmen nennt die EU die europäischen Struktur- und Investitionsfonds – insbesondere den Kohäsionsfonds und den Europäischen

78) Im Überblick auch zu den Regelungen in Bremen, Hessen, Niedersachsen und Rheinland-Pfalz *Knauff*, in: Rodi (Hrsg.), Handbuch Klimaschutzrecht, 2022, § 11, Rdnr. 5; *Kohlrausch*, ZUR 2020, 262, 265 f.; *Sina*, EurUP 2018, 314, 322.
79) Klimaschutzgesetz Baden-Württemberg (KSG BW) v. 23. 7. 2013, GBl. 2013, S. 229 i. d. F. des G. v. 12. 10. 2021, GBl. S. 837.
80) Berliner Klimaschutz- und Energiewendegesetz (EWG Bln) v. 22. 3. 2016, GVBl. S. 122 i. d. F. des G. v. 27. 8. 2021, GVBl. S. 989.
81) Energiewende- und Klimaschutzgesetz Schleswig-Holstein (EWKG SH) v. 7. 3. 2017, GVOBl. S. 124, i. d. F. des G. v. 2. 12. 2021, GVOBl. S. 1339.
82) Hamburgisches Klimaschutzgesetz (HmbGKlimG) v. 20. 2. 2020, HmbGVBl. 2020, S. 148.
83) Bayerisches Klimaschutzgesetz (BayKlimaG) v. 23. 11. 2020, GVBl. S. 598, 656.
84) Thüringer Gesetz zum Klimaschutz und zur Anpassung an die Folgen des Klimawandels (ThürKlimaG) v. 18. 12. 2018, GVBl. 2018, 816.
85) Gesetz zur Neufassung des Klimaschutzgesetzes Nordrhein-Westfalen vom 8. 7. 2021, GV. NRW S. 908; das ursprüngliche KSG NRW v. 29. 1. 2013, GV. NRW S. 29 hatte auch Aspekte der Klimaanpassung geregelt, s. *Fischer*, Grundlagen und Grundstrukturen eines Klimawandelanpassungsrechts, 2013, S. 19 f.
86) Klimaanpassungsgesetz Nordrhein-Westfalen (KlAnG) v. 8. 7. 2021, GV. NRW S. 893.
87) Ähnlich das Gesetzesziel gem. § 4a S. 1 KSG Baden-Württemberg die „unvermeidbaren Auswirkungen des Klimawandels … im Rahmen einer landesweiten Anpassungsstrategie durch vorsorgende Anpassungsmaßnahmen zu begrenzen".
88) Gesetzentwurf der Landesregierung Klimaanpassungsgesetz Nordrhein-Westfalen (KlAnG) v. 11. 3. 2021, LT-Drs. NRW 17/12977, S. 11.
89) Vgl. dazu *Knauff*, in: Rodi (Hrsg.), Handbuch Klimaschutzrecht, 2022, § 11, Rdnr. 13.
90) Dazu *Gerber/Hohmann*, VBlBW 2022, 97, 100.
91) Ähnlich das Berliner Energie- und Klimaschutzprogramm 2030, das ebenfalls zugleich Emissionsminderungs- und Anpassungsmaßnahmen enthält, vgl. Flaskühler, LKV 2018, 248, 250.
92) *Sina*, EurUP 2018, 314, 322.
93) Dazu *Flaskühler*, LKV 2018, 248, 250.
94) *Sina*, EurUP 2018, 314, 322; *Knauff*, in: Rodi (Hrsg.), Handbuch Klimaschutzrecht, 2022, § 11, Rdnr. 22.
95) Komm., Mitt. v. 24. 2. 2021, Ein klimaresilientes Europa aufbauen – die neue EU-Strategie für die Anpassung an den Klimawandel, COM(2021) 82 final, S. 11.

Fonds für regionale Entwicklung (EFRE), die Gemeinsame Agrarpolitik, das EU-Förderprogramm für Umweltschutzmaßnahmen LIFE und die im Kontext der Corona-Pandemie zur Abfederung von Krisenfolgen auf EU-Ebene etablierte Aufbau- und Resilienzfazilität.[96] Zudem möchte die EU die Kommunen beim Zugang zu klimaanpassungsrelevanten Datenbeständen und digitalen Infrastrukturen unterstützen.[97] Die Bundesregierung hat die Bedeutung der lokalen und regionalen Ebene etwa in der Deutsche Anpassungsstrategie an den Klimawandel hervorgehoben.[98] Klimaanpassung sei „vor allem eine Aufgabe für Kommunen und Regionen".[99]

Viele Gemeinden und Kreise haben bereits Klimaanpassungskonzepte verabschiedet oder bereiten solche vor.[100] Große Bedeutung hat dabei die Rückbindung an die Klimaanpassungsgesetzgebung des jeweiligen Bundeslandes. Verschiedene Landesgesetze enthalten ausdrückliche Vorgaben an die Klimaanpassungsplanung auf kommunaler Ebene. Gem. § 3 Abs. 1 i. V. m. § 2 Abs. 1 KlAnG NRW zählen neben der Landesregierung und weiteren Landesbehörden auch Gemeinden und Gemeindeverbände zu den „öffentlichen Stellen", die verpflichtet sind, die „negativen Auswirkungen des Klimawandels ... durch die Erarbeitung und Umsetzung von handlungsfeldspezifischen und auf die jeweilige Region abgestimmten Anpassungsmaßnahmen zu begrenzen". Das Klimaschutzgesetz Thüringen sieht in § 12 die Möglichkeit eigener Untersuchungen der Landkreise und Gemeinden zur „Verwundbarkeit durch Klimafolgen" als Grundlage individueller Anpassungskonzepte vor.

Auch bundesrechtliche Vorgaben sind bedeutsam, etwa Vorgaben zur Beachtung von Klimaanpassungsbelangen in der Bauleitplanung. Im BauGB gibt es bereits seit geraumer Zeit entsprechende Rechtsgrundlagen im Leitbild der „klimagerechten Stadtentwicklung".[101] Mit der Klimaschutz-Novelle 2011 zum BauGB[102] wurden die Ziele der Bauleitplanung in § 1 BauGB um die „allgemeine Klimaschutzklausel"[103] des § 1 Abs. 5 S. 2 BauGB ergänzt. Danach sollen die Bauleitpläne ausdrücklich auch dazu beitragen, die Klimaanpassung zu fördern. Ergänzend wurde § 1a Abs. 5 BauGB eingefügt, wonach bei der Aufstellung von Bauleitplänen den Erfordernissen des Klimaschutzes auch durch „Maßnahmen, ..., die der Anpassung an den Klimawandel dienen, Rechnung getragen werden [soll]" und dies in der Abwägung zu berücksichtigen ist.

Nach den Vorschriften der §§ 136 ff. BauGB zum Besonderen Städtebaurecht kann die Klimaanpassung von den Gemeinden zum Ziel städtebaulicher Sanierungsmaßnahmen gemacht werden. Maßgeblich hierfür sind gesetzliche Ergänzungen durch die sog. Innenentwicklungs-Novelle 2013 zum BauGB.[104] Gem. § 136 Abs. 2 Nr. 1 BauGB können Defizite bei der Klimaanpassung in einem Gebiet wegen der negativen Implikationen für die allgemeinen Anforderungen an gesunde Wohn- und Arbeitsverhältnisse oder an die Sicherheit der in ihm wohnenden oder arbeitenden Menschen einen städtebaulichen Missstand begründen, der zum Ausgangspunkt städtebaulicher Sanierungsmaßnahmen werden kann.[105] Gem. § 136 Abs. 4 S. 2 Nr. 1 BauGB sollen städtebauliche Sanierungsmaßnahmen dazu beitragen, dass „die bauliche Struktur in allen Teilen des Bundesgebiets nach den allgemeinen Anforderungen an den Klimaschutz und die Klimaanpassung sowie nach den sozialen, hygienischen, wirtschaftlichen und kulturellen Erfordernissen entwickelt wird".[106] Weiterhin können die Gemeinden Maßnahmen der Klimaanpassung auch aufgrund der Regelungen der §§ 171a bis 171d BauGB zum Stadtumbau umsetzen.[107]

## 7. Fazit und Ausblick

Mit dem Fortschreiten des Klimawandels entstehen im Mehr-Ebenen-System des Rechts zahlreiche Regelungen zur Anpassung an Klimaänderungen: Im völkerrechtlichen Pariser Abkommen, im EU-Klimagesetz, auf Bundesebene, auf Landesebene und in den Kommunen. Das wichtigste verbindende Element der Regelungen auf den verschiedenen rechtlichen Ebenen ist die gemeinsame Zielsetzung der Anpassung an nachteilige Auswirkungen von Klimaänderungen. Die übergeordnete Zielsetzung der Klimaanpassung wird regelmäßig mit Planungsgeboten verbunden, die zur nachfolgenden planerischen Festlegung von Maßnahmen zur Klimaanpassung verpflichten. Die Verknüpfung von Zielsetzung und Planungsgeboten – oft ergänzt um Vorgaben zum Monitoring, zur Einbeziehung von sachverständiger Expertise und zur Planfortschreibung – findet sich im Pariser Abkommen, im EU-Klimagesetz sowie im Recht der Bundesländer und empfiehlt sich auch als Element eines neu zu schaffenden Klimaanpassungsgesetzes des Bundes. Auf der Ebene der Anpassungsplanungen ist dann der Vielgestaltigkeit und Unterschiedlichkeit Rechnung zu tragen, die Klimaanpassungsbedarfe mit Blick auf Unterschiede von Naturräumen, Siedlungsstrukturen und Infrastrukturen haben können. Für die kommenden Jahre ist eine weitere Vertiefung und Ausdifferenzierung der Rechtsgrundlagen des Klimaanpassungsrechts zu erwarten. Das wichtigste Gesetzgebungsprojekt in Deutschland ist die im Koalitionsvertrag 2021 – 2025 vorgesehene Schaffung eines Klimaanpassungsgesetzes auf Bundesebene.

**Open Access.** Dieser Artikel wird unter der Creative Commons Namensnennung 4.0 International Lizenz veröffentlicht, welche die Nutzung, Vervielfältigung, Bearbeitung, Verbreitung und Wiedergabe in jeglichem Medium und Format erlaubt, sofern Sie den/die ursprünglichen Autor(en) und die Quelle ordnungsgemäß nennen, einen Link zur Creative Commons Lizenz beifügen und angeben, ob Änderungen vorgenommen wurden.
Die in diesem Artikel enthaltenen Bilder und sonstiges Drittmaterial unterliegen ebenfalls der genannten Creative Commons Lizenz, sofern sich aus der Abbildungslegende nichts anderes ergibt. Sofern das betreffende Material nicht unter der genannten Creative Commons Lizenz steht und die betreffende Handlung nicht nach gesetzlichen Vorschriften erlaubt ist, ist für die oben aufgeführten Weiterverwendungen des Materials die Einwilligung des jeweiligen Rechteinhabers einzuholen.
Weitere Details zur Lizenz entnehmen Sie bitte der Lizenzinformation auf http://creativecommons.org/licenses/by/4.0/deed.de.

Open Access funding enabled and organized by Projekt DEAL.

96) Komm., Mitt. v. 24.2.2021, Ein klimaresilientes Europa aufbauen – die neue EU-Strategie für die Anpassung an den Klimawandel, COM(2021) 82 final, S. 11.
97) Komm., Mitt. v. 24.2.2021, Ein klimaresilientes Europa aufbauen – die neue EU-Strategie für die Anpassung an den Klimawandel, COM(2021) 82 final, S. 11.
98) Deutsche Anpassungsstrategie an den Klimawandel, Beschluss des Bundeskabinetts v. 17.12.2008, S. 7, 15.
99) Fortschrittsbericht der Bundesregierung zur Deutschen Anpassungsstrategie an den Klimawandel, Stand 16.11.2015, Stand 12.5.2022, abrufbar unter https://www.bmuv.de/fileadmin/Daten_BMU/Download_PDF/Klimaschutz/klimawandel_das_fortschrittsbericht_bf.pdf, S. 26.
100) Vgl. *Hasse/Willen/Baum/Bongers-Römer/Pichl/Völker*, Umfrage Wirkung der DAS für die Kommunen. Teilbericht, Climate Change 1/2019, S. 34 f., Stand 18.5.2022, abrufbar unter https://www.umweltbundesamt.de/sites/default/files/medien/1410/publikationen/2019-01-21_cc_01-2019_umfrage-das.pdf.
101) Vgl. *Battis/Krautzberger/Mitschang/Reidt/Stüer*, NVwZ 2011, 897 ff.; zur Rechtslage nach dem BauGB 2004 *Schmidt*, NVwZ 2006, 1354 ff.
102) Gesetz zur Förderung des Klimaschutzes bei der Entwicklung in den Städten und Gemeinden v. 22.7.2011, BGBl. I S. 1509.
103) Begriff u. a. bei *Mitschang*, ZfBR 2020, 613, 614.
104) Gesetz zur Stärkung der Innenentwicklung in den Städten und Gemeinden und weiteren Fortentwicklung des Städtebaurechts v. 11.6.2013, BGBl. I S. 1548.
105) Näher *Mitschang*, ZfBR 2020, 613, 615 f.
106) Näher *Mitschang*, ZfBR 2020, 613, 616 f.; *Groth/Bender/Groth*, ZfU 2021, 385, 395.
107) *Mitschang*, ZfBR 2020, 613, 620 ff.; *Groth/Bender/Groth*, ZfU 2021, 385, 396 f.
